# Extraskeletal multiple myeloma presenting with an atrial mass: a case report and a review of the literature

**DOI:** 10.1186/1752-1947-6-236

**Published:** 2012-08-10

**Authors:** Federica Vigo, Patrizia Ciammella, Riccardo Valli, Elisabetta Cagni, Cinzia Iotti

**Affiliations:** 1 Department of Advanced Technologies, Radiation Oncology Unit, Arcispedale Santa Maria Nuova - IRCCS, Reggio Emilia, Italy; 2 Department of Oncology, Pathology Unit, Arcispedale Santa Maria Nuova - IRCCS, Reggio Emilia, Italy; 3 Medical Physics Department, Arcispedale Santa Maria Nuova - IRCCS, Reggio Emilia, Italy

## Abstract

**Introduction:**

Extraskeletal presentation at diagnosis or during the course of multiple myeloma is a rare event. The prognosis is usually very poor. At the moment there is no agreed gold standard for the treatment of this presentation.

**Case presentation:**

A 79-year-old Caucasian woman was treated at our hospital for right atrial myeloma localization. Our patient showed the following signs and symptoms of congestive heart failure: dyspnea, hypotension, cyanosis and facial edema. Surgery was not considered feasible due to the extent of the disease. Our patient underwent external-beam radiation therapy using an intensity modulated technique, thus obtaining a persistent complete remission. Our patient has been in continuous complete local remission for 25 months since the end of radiotherapy.

**Conclusion:**

The role of radiotherapy is not defined in multiple myeloma with extraskeletal presentation. Our regimen seems to be effective in controlling the disease in this patient.

This case report adds to the existing literature as it describes an unusual presentation of the disease and a new therapeutic approach to this rare presentation of multiple myeloma.

## Introduction

Multiple myeloma (MM) is a malignant disorder of the plasma cells that is commonly associated with bone marrow plasmacytosis and usually restricted only to the bone marrow. Extraskeletal (ES) localization at diagnosis or during the course of MM is a rare event with a very aggressive course. The prognosis is extremely poor, especially when the diagnosis of ES localization is concurrent with the diagnosis of MM [1]. Patients presenting with ES involvement at diagnosis have significantly shorter progression-free survival compared with other patients with MM (18 months versus 30 months, P=0.003), whereas the median overall survival is not statistically different between the two groups (36 and 43 months, respectively, P=0.36) [2].

Intracardiac localization is extremely rare, with only a few case reports [2-8].

We present the case of a patient with a right atrial mass that led to signs and symptoms of congestive heart failure.

## Case presentation

A healthy 79-year-old Caucasian woman was referred to our hospital with a diagnosis of stage III immunoglobulin A-lambda MM with numerous osteolytic areas (bilateral iliac wings, T3 to T4) and cardiologic symptoms of pulmonary embolism. Computed tomography (CT) of her chest documented the presence of a right atrial mass (42mm × 53mm), confirmed by subsequent magnetic resonance imaging (MRI) and fluorine-18-fluorodeoxyglucose positron emission tomography (F18-FDG PET). A transesophageal echocardiogram revealed a large pericardial effusion with evidence of cardiac tamponade and a large right atrial mass encasing her interatrial septum and extending into her left atrium. Histologic specimens of a bone marrow biopsy demonstrated the presence of tiny aggregates of atypical plasma cells with asynchronous morphology, positive for cluster of differentiation 138.

Our patient was treated with four cycles of bortezomib, four cycles of melphalan and prednisone and another four cycles of bortezomib, achieving a lessening of the symptoms and a stability of the cardiac disease. Pharmacological toxicity was not significant.

Nine months later, our patient developed a painful bone relapse. The pain was severe, constant and localized primarily in her sacrum and the right iliac wing. The pain increased to some extent with movement. Our patient received local radiotherapy on both sites (2000cGy/5 fractions), achieving a reduction in symptoms and an improvement of her performance status.

One year after the initial diagnosis, our patient presented to our emergency department because of the new appearance of signs and symptoms of congestive heart failure: dyspnea, hypotension, cyanosis and facial edema.

**Figure 1:**
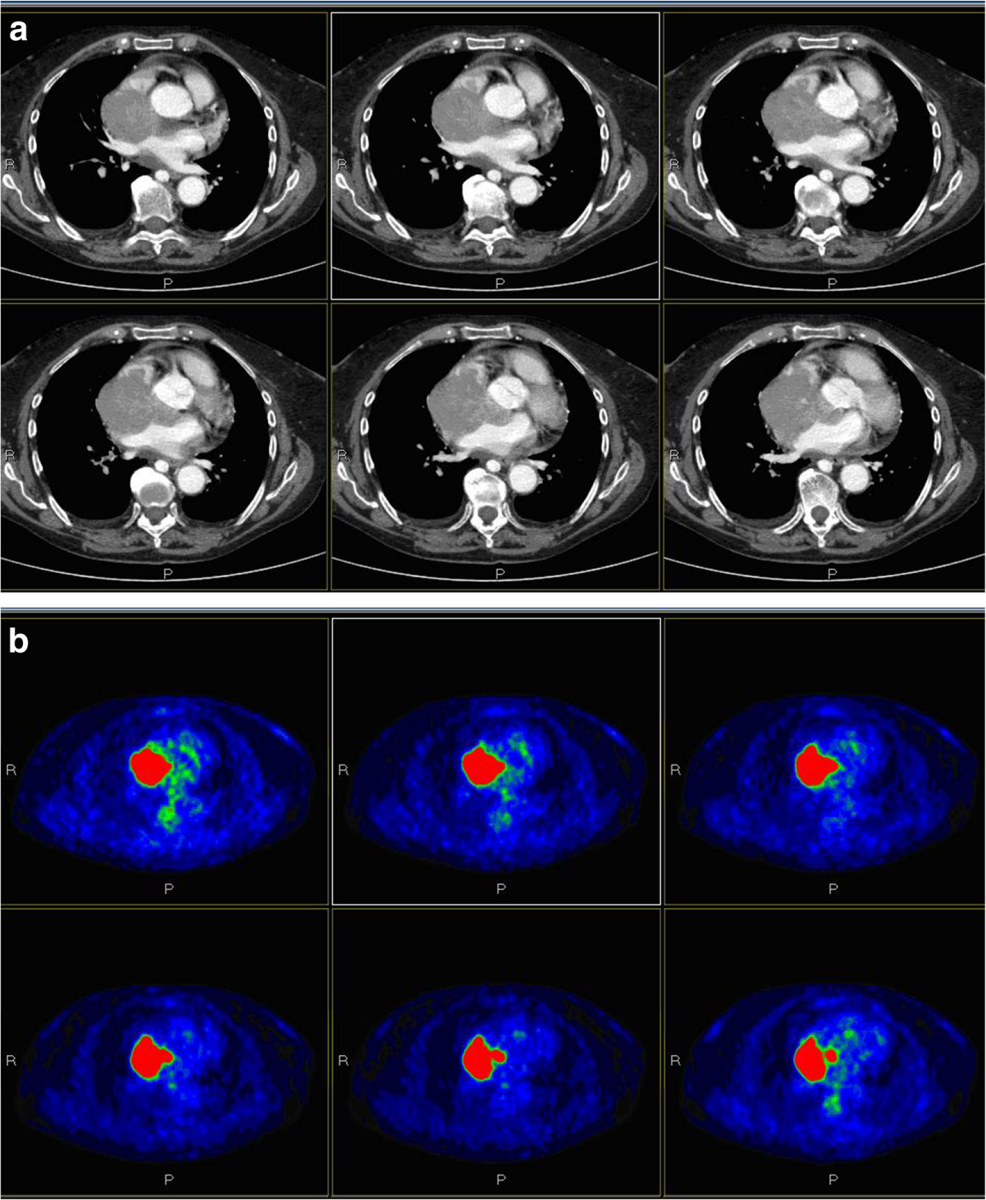
**Radiographic images of the mass.** ( **A**) Computed tomography of the chest documenting the atrial mass; ( **B**) fluorine-18-fluorodeoxyglucose positron emission tomography confirming this data.

CT of her chest revealed an increase in the size of the mass (78mm × 84mm), which appeared to infiltrate her right pulmonary vein and superior vena cava for approximately 21mm, causing neoplastic thrombosis of her right subclavian vein (Figure 1A). These findings were confirmed by echocardiography and F18-FDG PET (Figure 1B).

Restaging examinations documented stable bone disease.

Our patient was evaluated by a surgeon for possible surgery on her right atrium, but she was considered inoperable due to the extent of the disease. The diagnosis of atrial localization of malignant plasmacytoma was confirmed via examination of a biopsy specimen. In order to obtain rapid control of the intracardiac disease, radiation to the cardiac mass was started.

External-beam radiation, totaling 3000cGy in 10 daily fractions of 300cGy, each with 6mV photons, was delivered using the intensity modulated radiation therapy (IMRT) technique.

Our patient was immobilized in the supine position with a wing board and target localization was accomplished using CT simulation. The volumes of interest were identified on each axial CT slice. The gross tumor volume was defined as the gross extent of the tumor shown by imaging (CT, MRI and PET); the clinical target volume was defined as the gross tumor volume plus a margin for potential microscopic spread (5mm); and, in order to account for organ motion and patient setup errors, the planning target volume was defined by adding a 1 cm margin to the clinical target volume. Treatment planning was performed using an inverse planning algorithm in a tomotherapy planning system and our patient was treated with an IMRT delivered with tomotherapy.

The treatment was generally well tolerated; our patient showed no symptoms suggestive of radiation-induced toxicity. Her symptoms immediately improved with a decrease in generalized edema and improved performance status. A transesophageal echocardiogram performed three weeks from the end of the radiotherapy confirmed a significant decrease in tumor size in her right atrium.

Due to our patient’s age and good condition, the medical staff chose a ‘wait and see’ approach. Three months later, repeat chest and abdominal CT scans showed complete remission of the cardiac disease, but progressive bone disease.

The worsening of the general conditions of our patient, due to other causes, meant that she was subjected to supportive care for two months. After the improvement of her health status, our patient started maintenance chemotherapy with lenalidomide and she is currently tolerating it well.

**Figure 2:**
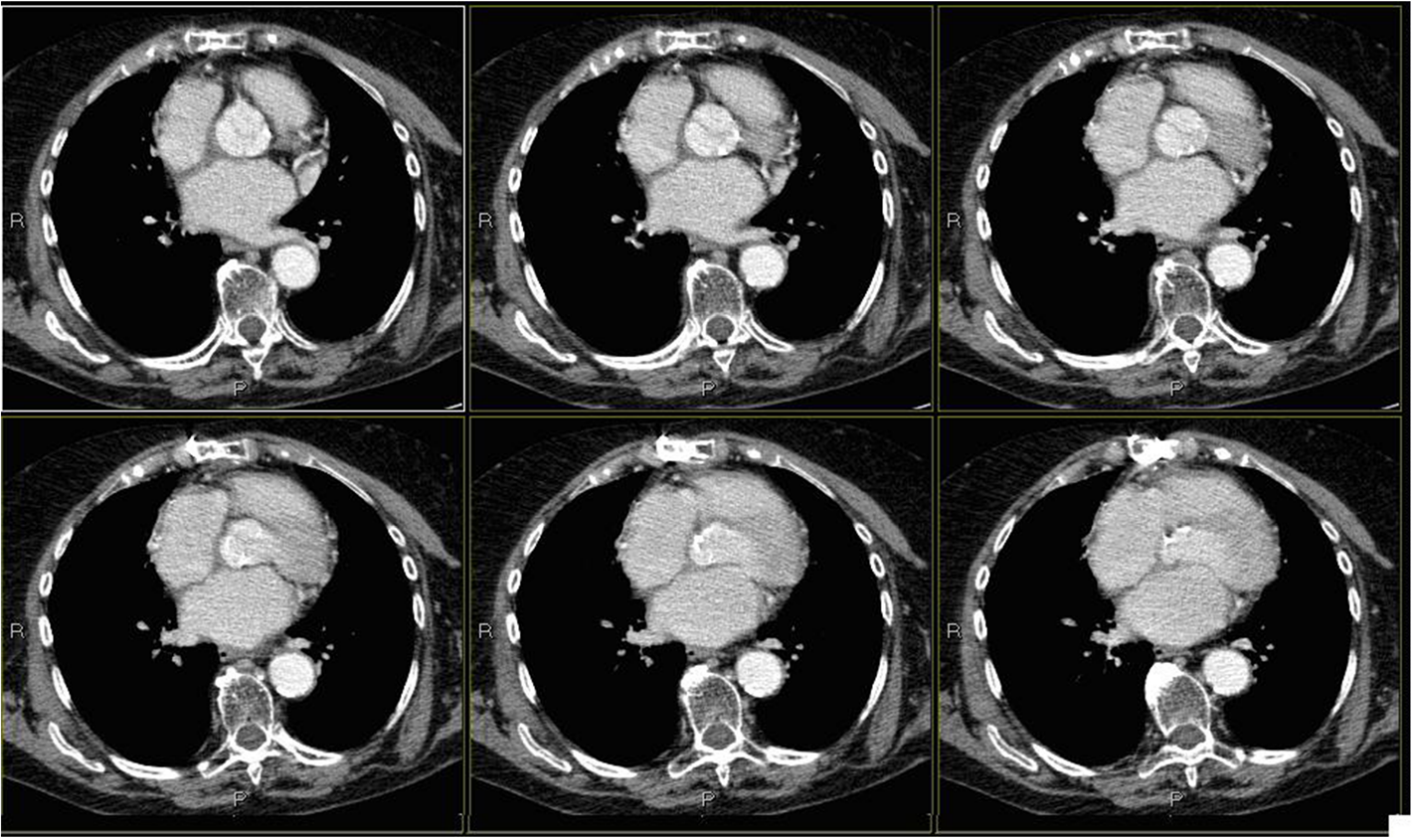
**Computed tomography of the chest performed one year after the end of radiotherapy.** Image describes total disappearance of the lesion.

In a new CT scan of her chest done eighteen months after the end of the radiotherapy (Figure 2), complete remission of the cardiac disease was confirmed.

Currently, our patient is generally in good condition and shows no significant cardiac symptoms. Recently performed chest and abdominal CT documented stability of the disease in known locations, but highlighted the emergence of an axillary mass, for which we are programming a new radiation treatment in view of her good response to previous radiotherapy.

## Discussion

There are few previous case reports of intracardiac malignant plasmacytomas (Table 1); for this reason, experience with the treatment of this involvement is limited. Furthermore, in some cases, the diagnosis was made during autopsy [1,6].

**Table 1:** Review of the literature

**Reference**	**Extramedullary localization**	**Diagnostic test**	**Local therapy**	**Outcome portion**	**Cause of death**
Goldberg and Mori [3]	Pericardial effusion and cardiac tamponade	Autopsy	NR	Death and postmortem diagnosis of pericardial involvement	Heart failure
Garrett *et al*. [4]	Pericardial and myocardial involvement and cardiac tamponade	Chest X-ray	Transthoracic pericardiocentesis	No medical therapy for heart involvement	Heart failure
Imamura *et al*. [5]	Pleural and pericardial effusion	NR	Pericardiocentesis, intrapericardial injection of OK-432, RT (1400rad), peplomycin, vincristine and prednisolone	CR	Death seven months after diagnosis for progression of systemic disease
Mitchell *et al*. [6]	Pericardial effusion and substantial hypertrophy of the right and left ventricular walls, infiltrative cardiomyopathy	Echocardiogram	Bleomycin 20mL in 30mL of normal saline solution introduced into the pericardial space	No recurrent pericardial effusion	36 hours after his last echocardiogram, the patient became acutely hypoxemic and died suddenly (massive pulmonary embolism) -no autopsy
Ueda *et al*. [8]	A-V sulcus between the left atrium and left ventricle (diameter 3cm) Cardiac tamponade	TEE	Cisplatin-betamethasone into the pericardial cavity	CR	Death from bacterial pneumonia 182 days after the first admission - no autopsy
Champeaux *et al*. [9]	Myocardium and coronary vessels	Autopsy	NR	NR	Respiratory failure
Owens *et al*. [10]	Pericardial effusion and large mass lesions in the left and right atria. Cardiac tamponade	Echocardiography, chest radiography and CT of the heart	Drainage of the effusion, RT to the heart (30Gy/10fr over two weeks with 6mV photons)	Almost complete tumor regression	Alive with disease
Zeiser *et al*. [11]	Pericardial and pleural effusion	Echocardiography, CT of the thorax	High-dose systemic dexamethasone	Stable disease for six weeks	Death from pneumonia
Songul *et al*. [12]	Left lobe and isthmus of thyroid, bilateral pleural effusion and a 1cm pericardial effusion around the left ventricle.	Chest radiography, CT	Chemotherapy, RT and supportive measures.	Death	Disease progression
Franzese *et al*. [13]	Pericardium infiltration	Chest radiography, CT and echocardiogram.	Surgical resection of the intrapericardial mass	NR	NR
Paulus *et al*. [14]	Large pericardial effusion, large right atrial mass encasing the interatrial septum extending into the left atrium, cardiac tamponade	Chest radiography, TEE, MRI of the chest, biopsy of the atrial mass	Pericardiocentesis, high-dose dexamethasone, bortezomib and lenalidomide RT to the cardiac mass (20Gy/10fr with 6mV photons using an Anterior-Posterior technique Consolidation unspecified chemotherapy (bortezomib, cyclophosphamide, dexamethasone)	Significant decrease in tumor size in the right atrium and the aortic root	NR

CT: computed tomography; MRI: magnetic resonance imaging; NR: not reported; RT: radiotherapy; TEE: transesophageal echocardiogram; OK-432 is an immunomodulator derived from Streptococcus; CR: complete remission.

Interestingly, the right atrium seems to be the predominant location of plasmacytomas involving the heart. Although its exact etiology is unknown, the presence of cell surface adhesion molecules on the malignant plasma cell and their interaction with permissive growth factors on the endothelial lining of the heart have been considered as contributing factors [7].

The most optimal therapeutic strategy for intracardiac malignant plasmacytoma is not well defined. Treatment options include, when possible, surgical resection or intrapericardial administration of bleomycin, cisplatin, or betamethasone with only transitory effects, or radiation therapy (with different doses).

Until now, notwithstanding the wide range of approaches available, there is no unanimous agreement on the proper mode of treatment for intracardiac malignant plasmacytoma.

If we analyze the studies reported in Table 1, it is quite evident how the radiation therapy, in cases in which it was administered, especially when associated with systemic therapy [5,10,14], has resulted in a better clinical and instrumental response than the other treatments, especially considering the better toxicity profile of radiation therapy compared with surgery. Another possible alternative would seem to be the intrapericardial injection of different drugs, that, in the few cases reported in literature [5,6,8], seem to have allowed reasonable local control.

Our patient’s clinical presentation of congestive heart failure was initially a result of tamponade and also a result of the anatomic location of the MM in her right atrium. In this case, the radiation treatment was useful, not only to reduce the bone pain, but also to decrease the cardiac disease burden with an immediate improvement in our patient’s symptoms and performance status.

After a follow-up of 25 months, our patient is alive with good general condition and she has no limitations to her daily activities, considering her age and maintenance therapy.

This result leads us to suggest the possibility to obtain not only control of the symptoms, but also a beneficial effect in delaying disease progression.

## Conclusions

ES tumors are a rare manifestation of MM, with a cumulative incidence of 4.6% of MM cases. The best treatment strategy for these tumors is not yet well defined. The role of radiotherapy, which is the standard treatment for a solitary plasmacytoma, is much less defined in MM with ES disease, where radiotherapy is usually associated with systemic treatment with chemotherapy or novel agents.

Studies aimed at defining the best therapeutic strategy for ES MM are needed.

## Consent

Written informed consent was obtained from the patient for publication of this manuscript and accompanying images. A copy of the written consent is available for review by the Editor-in-Chief of this journal.

## Competing interests

The authors declare that they have no competing interests.

## Authors’ contributions

FV has contributed to the clinical evaluation of the patient and was a major contributor in writing the manuscript; PC was the radiation oncologist of reference of the patient and contributed in writing the manuscript; CI, the Director of the Radiation Oncology Unit, has contributed to patient management and therapeutic decisions; RV analyzed and interpreted the patient data from a histological point of view; EC drew up the plan of radiotherapy. All authors read and approved the final manuscript.
